# The Outcomes of Acute Coronary Syndrome in Patients Suffering From Schizophrenia: A Systematic Review

**DOI:** 10.7759/cureus.16998

**Published:** 2021-08-08

**Authors:** Hanan Hannoodee, Mahmoud Al Khalili, Nyein Wint Yee Theik, Oluwatimilehin E Raji, Priya Shenwai, Rutul Shah, Sahithi Reddy Kalluri, Tinaz H Bhutta, Safeera Khan

**Affiliations:** 1 Internal Medicine, California Institute of Behavioral Neurosciences & Psychology, Fairfield, USA; 2 Internal Medicine, M.P. Shah Government Medical College, Jamnagar, IND

**Keywords:** acute coronary syndrome, myocardial infarction, schizophrenia, outcomes, acs, review article

## Abstract

Acute coronary syndrome (ACS) is a principal cause of mortality and morbidity worldwide. Recent studies have suggested poorer outcomes in ACS patients who have a concurrent diagnosis of schizophrenia as compared with those without. However, the degree of interplay between schizophrenia and ACS remains poorly understood. For this reason, we conducted a systematic review on ACS outcomes in patients with schizophrenia by following the Preferred Reporting Items for Systematic Reviews and Meta-Analyses (PRISMA) guidelines. We collected relevant data from PubMed, Cochrane Library, PubMed central, Jisc Library Hub Discover, and the National Library of Medicine (NLM) and performed a thorough quality appraisal. Fourteen shortlisted, relevant studies were meticulously reviewed. Mortality and major adverse cardiac events (MACE), bleeding, and stroke were more prevalent in patients with a schizophrenia diagnosis compared to those without. Additionally, schizophrenia patients received suboptimal care and follow-up when compared to patients without a psychiatric diagnosis. Clinicians need to be aware that patients with schizophrenia have worse outcomes following ACS which may relate to biological, health care, or patient-related factors.

## Introduction and background

Acute coronary syndrome (ACS) occurs as a result of sudden rupture of unstable atherosclerotic plaques with subsequent thrombus formation in the coronary arteries and loss of blood flow to the heart muscle [1]. It carries a significant burden of mortality and morbidity (heart failure, re-infarcts, arrhythmia), which may be reduced by the timely use of revascularization procedures and evidenced-based cardioprotective therapies [2-5]. Interestingly, mortality risk in patients with ACS has been suggested to be higher in patients with a previous diagnosis of schizophrenia [6].

Schizophrenia is a severe mental illness that causes considerable disability and higher rates of mortality with an estimated 15-25 years decrease in life expectancy in this group [7-8]. Odds of coronary heart disease were found to be higher in schizophrenia patients compared to the general population (odds ratio (OR)=1.51; 95% CI: 1.47-1.55) [9]. Patients who have a diagnosis of schizophrenia are considered to be more vulnerable to cardiovascular diseases (CVD) than mentally healthy patients due to the high prevalence of CVD risk factors, such as obesity, metabolic disorder, diabetes, and smoking, in those patients [10-11]. Moreover, it has been shown that there is inequality in the management of ACS between patients with schizophrenia and mentally healthy patients with lower rates of revascularization and cardioprotective therapy prescriptions [12-16]. As a consequence, a number of recent studies have explored whether ACS outcomes are worse in patients with schizophrenia using various study designs [17].

There is variability and inconsistency in the results of the reviewed studies that are exploring the mortality and morbidity risk in schizophrenic patients who are diagnosed with ACS. The relationship is not fully explored yet, and a significant knowledge gap is still present when managing schizophrenia patients with ACS. Therefore, we are conducting this systematic review to evaluate the major outcomes of ACS in patients with a pre-existing schizophrenia diagnosis as compared to those without.

## Review

Methods

We conducted a systematic review following Preferred Reporting Items for Systematic Reviews and Meta-Analyses (PRISMA) guidelines [18].

Data Collection and Search Strategy

An electronic search of published studies was conducted to identify relevant articles on the following databases: PubMed, Cochrane Library, PubMed Central, and Medline, with some other relevant articles extracted from Jisc Library Hub Discover and National Library of Medicine (NLM).

The PubMed search strategy was based on an algorithm made with the use of MeSH terms (Medical Subject Headings) using the following keywords:

Schizophrenia OR ( "Schizophrenia/complications"[Majr] OR "Schizophrenia/epidemiology"[Majr] OR "Schizophrenia/mortality"[Majr] OR "Schizophrenia/physiopathology"[Majr] OR "Schizophrenia/prevention and control"[Majr] OR "Schizophrenia/statistics and numerical data"[Majr] ) AND Acute coronary syndrome OR ACS OR( "Acute Coronary Syndrome/epidemiology"[Majr] OR "Acute Coronary Syndrome/etiology"[Majr] OR "Acute Coronary Syndrome/history"[Majr] OR "Acute Coronary Syndrome/mortality"[Majr] OR "Acute Coronary Syndrome/physiopathology"[Majr] OR "Acute Coronary Syndrome/prevention and control"[Majr] OR "Acute Coronary Syndrome/statistics and numerical data"[Majr] ) OR myocardial infarction OR MI OR (( "Myocardial Infarction/epidemiology"[Majr] OR "Myocardial Infarction/etiology"[Majr] OR "Myocardial Infarction/history"[Majr] OR "Myocardial Infarction/mortality"[Majr] OR "Myocardial Infarction/physiopathology"[Majr] OR "Myocardial Infarction/prevention and control"[Majr] OR "Myocardial Infarction/statistics and numerical data"[Majr] )).

Study Selection and Eligibility Criteria

All articles were transferred to a worksheet (Excel, Microsoft Inc, Redmond, WA). Duplicate articles were excluded. Each article was screened by title and abstract and was either included or excluded for relevance. All articles included were further evaluated by reviewing the full article text to exclude any irrelevant articles.

The inclusion criteria include all study types and designs from inception to the present day that are related to the topic of ACS in patients with schizophrenia. All population groups were included in this study. Only full-text articles were used, all of which were published in peer-reviewed journals. Any gray literature or unpublished articles were excluded. Non-English articles were also excluded.

 *Risk of Bias Assessment*

 The quality of included studies was assessed by the following tools:

a) Newcastle-Ottawa checklist - observational studies; b) AMSTAR checklist for systematic review and meta-analysis; c) Scale for the Assessment of Narrative Review Articles (SANRA) checklist - traditional review articles.

Results

Study Identification and Selection Results

**Figure 1:**
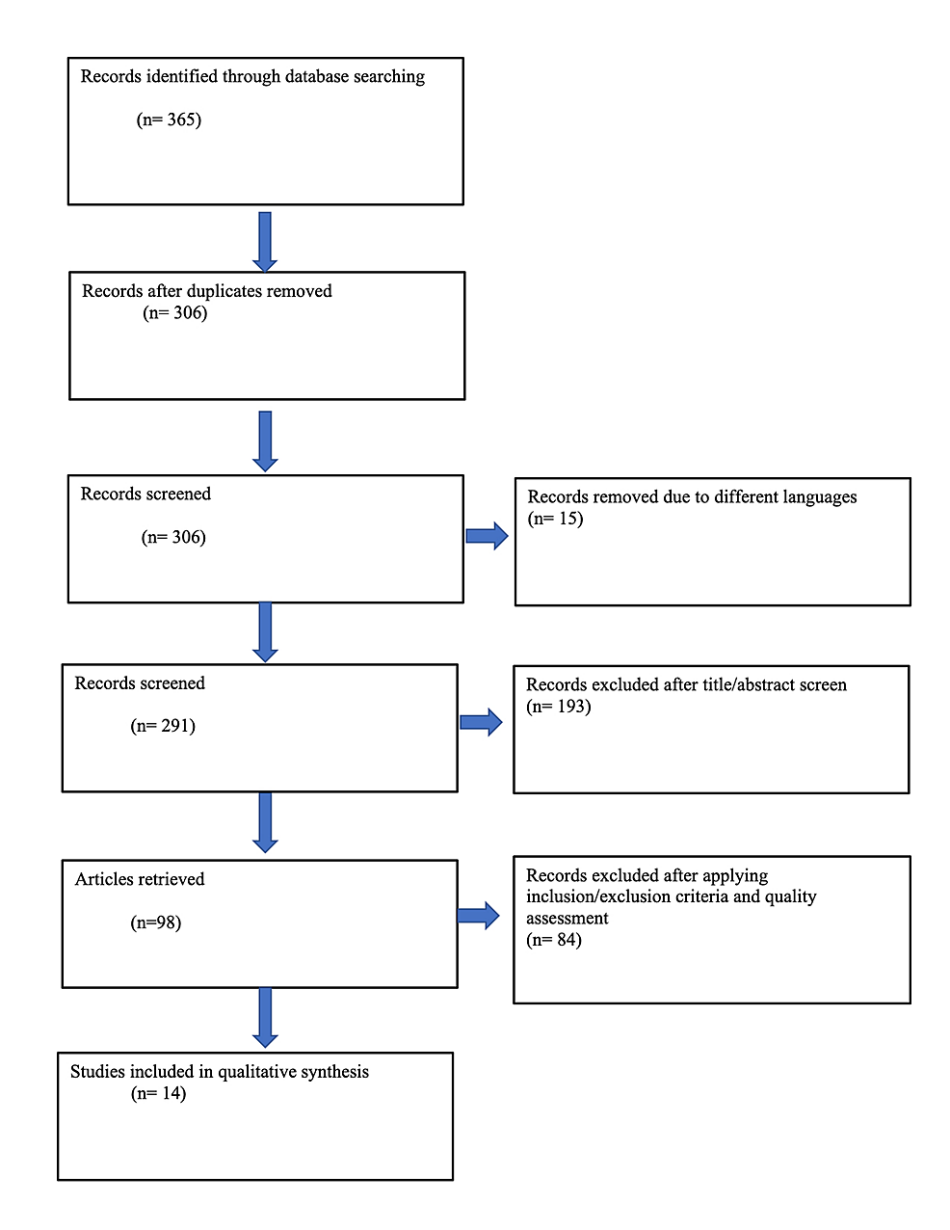
PRISMA flow diagram outlining the systematic review process PRISMA: Preferred Reporting Items for Systematic Reviews and Meta-Analyses

The initial search yielded 368 published articles. Duplicates (n=62) and non-English articles (n=15) were removed, leaving 291 articles to be screened by title and abstract for relevance. Of these, 193 articles were excluded for not being relevant. The full texts of the remaining 98 articles were thoroughly read, and 73 of them were excluded after inclusion/exclusion criteria were applied. Lastly, after a meticulous quality assessment, 11 articles were further excluded, and finally, 14 articles were included in the review. Figure 1 depicts the search process in the form of a PRISMA flow diagram.

Study Characteristics

In total, 14 articles were included in this review. The condition investigated was the outcomes of acute coronary syndrome in schizophrenia patients. Information about mortality rates from ACS in schizophrenia patients was found in these articles, of which 13 were observational or cohort studies and one was a meta-analysis. The study sample sizes ranged from 188 patients to 23,582 patients with a study duration of one year to 36 years. Four observational studies demonstrated additional information about major comorbidities, including major adverse cardiac events (MACE): re-infarction, stroke, and death and bleeding.

Main Findings

Fourteen studies that looked into the mortality outcome in schizophrenia patients with a diagnosis of ACS were summarized in Table 1. The studies employed various statistical methods that mostly showed an increased risk of mortality with an odds ratio (OR)/hazard ratio (HR) ranging from 1.1 to 15.47. Two studies showed no difference in 30 days mortality [19-20].

**Table 1:** Mortality outcome in schizophrenia patients with ACS aHR = adjusted hazard ratio; RR = relative risk; HR = hazard ratio; OR = odd Ratio; CI = confidence interval; MRR = mortality rate ratio; aOR = adjusted odd ratio.

	Author, year, reference	Country	Study design	Influence on mortality	Outcome
1	Attar R. 2020, [16]	Sweden	Observational follow-up	Increased	aHR 2.38 (95% CI 1.84-3.09); p< 0.005
2	Shao M. 2020, [6]	-	Meta-analysis	Increased	RR 1.66 (95% CI 1.33-2.09); p< 0.001
3	Hauck T. S. 2020, [21]	Canada	Cohort population-based	Increased	HR 1.55 (95% CI 1.37-1.77)
4	Chang W. C. 2020, [22]	Hong Kong	Cohort population-based	Increased	30-day mortality OR 1.99 (95% CI 1.65- 2.39). 1-year mortality OR 2.13 (95% CI 1.79-2.54)
5	Attar R. 2019, [23]	Denmark	Cohort population-based	Increased	HR 2.54 (95% CI 2.22-2.90)
6	Mohamed M. O. 2019, [24]	United States	Retrospective observational	Increased	OR 1.10 (95% CI 1.04-1.16)
7	Westman J. 2018, [25]	Sweden	Cohort study	Increased	MRR 2.62 (95% CI 2.49–2.75)
8	Kugathasan, Pirathiv; 2018, [19]	Denmark	Cohort study	Increased long term mortality No difference in 30-day mortality	aHR 15.47 (95% CI 12.62-18.96)
9	Protty M. B. 2017, [26]	United Kingdom	Retrospective cohort	Increased	HR 1.38 (95% CI 1.24-1.54); p< 0.05
10	Schulman-Marcus J. 2016, [27]	United States	Observational follow-up	Increased	OR 1.31 (95% CI 1.24-1.40); p< 0.001
11	Bodén R. 2015, [28]	Sweden	Cohort study	Increased	30-day mortality OR 2.58 (95% CI 1.88-3.54). 1-year mortality OR 2.55 (95% CI 1.98-3.29)
12	Wu S. I. 2013, [29]	Taiwan	Case-control	Increased	OR 2.68 (95% CI 1.73-4.15)
13	Kurdyak P. 2012, [30]	Canada	Retrospective cohort	Increased	30-day mortality aOR 1.56 (95% CI 1.08-2.23); p=0.02
14	Druss B. G. 2000, [20]	United States	Retrospective cohort	No difference in 30-day mortality	30-day mortality RR 0.95; p .18

Upon further review, we were able to identify nine studies that looked more in-depth at the mortality rates in schizophrenic patients after ACS (Table 2). They were adjusted for different variables in an attempt to remove any confounding bias. Some of these studies noted large increases in the HR of death even after adjusting for factors like age, sex, smoking, comorbidities, previous coronary angiography (CAG) and previous percutaneous coronary intervention (PCI), discharge medication, and treatment with CAG and PCI. However, a study by Druss BG et al. noted only modest changes after adjusting for percutaneous transluminal coronary angioplasty (PTCA) and coronary artery bypass graft (CABG) [20]. Adjusting for time to revascularization as expected resulted in improvement in mortality HR since many schizophrenic patients tend to have delays in receiving revascularization procedures [21].

**Table 2:** Demonstrates changes in mortality rates with adjustment of different criteria HR = hazard ratio; OR = odd ratio; CI = confidence interval; CAG = coronary angiography; PCI = percutaneous coronary intervention; AMI = acute myocardial infarction; COPD = chronic obstructive pulmonary disease; RR = relative risk; PTCA = percutaneous transluminal coronary angioplasty; CABG = coronary artery bypass graft

No	Author, reference	Unadjusted ratio	Adjusted ratio	Criteria adjusted
1	Attar R. et al [16]	5-year mortality HR 1.44 (95% CI 1.31-1.59)	HR 2.38 (95% CI 1.84-3.09)	Age, sex, smoking, comorbidities, previous CAG and previous PCI, discharge medication, and treatment with CAG and PCI.
2	Hauck T. S. et al [21]	Unavailable	HR 1.38 (95% CI 1.20-1.58)	Time-varying revascularization
3	Chang W. [22]	30-day mortality OR 1.67 (95% CI 1.40-1.98) 1-year mortality OR 1.67 (95% CI 1.44-1.95)	OR 1.99 (95% CI 1.65-2.39) 1-year mortality OR 2.13 (95% CI 1.79-2.54)	Age, sex, calendar-year period of index admission, and medical comorbidities.
4	Kugathasan P. [19]	HR 18.61 (95% CI 16.31-21.24)	HR 15.47 (95% CI 12.62-18.96)	Age at entry, gender, calendar year, diabetes, hypertension, hyperlipidemia, and COPD.
5	Schulman-Marcus J. [27]	In-hospital mortality OR 1.41 (95% CI 1.35-1.47)	OR 0.85 (95% CI 0.70-1.02)	Age, gender, race, payer, hospital characteristics, co-morbidities, previous revascularization, and cardiogenic shock.
6	Bodén R. [28]	Unavailable	30-day mortality OR 2.58 (95% CI 1.88-3.54) 1-year mortality OR 2.55 (95% CI 1.98-3.29)	Age, gender, smoking, diabetes, previous hypertension, heart failure, stroke, peripheral vascular disease, infarction type, Killip classification, biomarker level, acute treatments, and secondary preventative drugs.
7	Wu S. I. [29]	Unavailable	In-patient mortality OR 2.68 (95% CI 1.73-4.58)	Demographic characteristics (age at first AMI, level of income, and level of urbanization), medical disorders, cardiac history, hospital properties, inpatients complications, and any catheterization or revascularization receipt
8	Kurdyak P. [30]	OR 1.64 (95% CI 1.15-2.32)	OR 1.56 (95% CI 1.08-2.23)	Age, sex income, hospitalization length of stay, comorbidities (ambulatory diagnostic groups), rurality and cardiologist and primary care visits in the year prior to incident AMI.
9	Druss B. G. [20]	Unavailable	30-day mortality RR 0.95; P 0.18	Demographic variables, cardiac risk factors, and history, admission characteristics, left ventricular function availability of technology, and transfer status plus PTCA and CABG.

Discussion

This review systematically analyzes the existing literature on how a diagnosis of schizophrenia affects rates of mortality and morbidity in patients with ACS and what factors make those patients more susceptible to poorer outcomes. Although the results of the studies were not always in agreement, this review found a clear pattern of higher mortality in patients with schizophrenia who suffer from ACS when compared with psychiatric healthy patients as will be discussed below. In addition, patients with schizophrenia represent a multi-morbid cohort with a higher cardiovascular risk and a more significant burden of comorbidities than those without schizophrenia. We therefore also discuss inequality in the care provided to patients with schizophrenia which may explain some of our findings.

Mortality Risk in Patients With Schizophrenia and ACS

Mortality rates in schizophrenia patients with ACS did not decrease over the last two decades in spite of the general trend of decreasing mortality rates from ACS in patients worldwide [6,31]. This is described by Westman J et al. through a national registry study (1987-2010) in Sweden which found that coronary heart disease and cerebrovascular disease were the two most frequent cardiovascular causes of death in patients with schizophrenia with ACS being responsible for more than half the deaths from coronary heart disease in those patients (mortality rate ratio (MRR) 2.62; 95% CI 2.49-2.75) [25]. Indeed, in a large cohort study of ACS patients reported by Pirathiv et al., patients with schizophrenia had increased rates of mortality in the 36-year study period (1980-2015) compared with ACS patients without schizophrenia (hazard ratios (HR) 15.47; 95% CI 12.62-18.96) [19]. A more contemporary study from the UK by Protty MB et al. reported a similar conclusion but with a more modest increase in mortality over their study period 2004-2014 (HR 1.38; 95% CI: 1.24-1.54) [26]. Similar findings were reported by others as demonstrated in Table 1.

A diagnosis of schizophrenia has drastic consequences on the patients, not only on long-term post-ACS mortality but also on short-term and inpatient mortality. That was described by some authors who specifically investigated the 30-day mortality rate in schizophrenia patients with ACS. Kurdyak P et al. [30], Boden R et al. [28], and Hauck TS et al. [21] demonstrated a noteworthy rise in the 30-day mortality rate in those patients with an adjusted odds ratio (aOR) of (1.56; 95% CI 1.08-2.23), (2.58; 95% CI 1.88-3.54), and (1.55; 95% CI 1.37-1.77), respectively. Furthermore, Chang WC described similar results of higher 30-day mortality (OR 1.99; 95% CI 1.65-2.39) although they derived this cohort study group from patients who were diagnosed with psychotic disorders and not only schizophrenia [22]. Two studies looked more specifically at the mortality risk during the ACS event hospitalization. These studies were done by Schulman-Marcus J et al. and Wu SI et al., who noted higher inpatient mortality in patients with schizophrenia with OR 1.31 (95% CI 1.24-1.40) and 2.68 (95% CI 1.73-4.15), respectively [27,29]. Despite these reports, however, a number of studies demonstrated the conflicting findings of no difference in 30-day mortality from ACS in patients with schizophrenia as compared to those without [19]. These studies may have been limited however by smaller sample size (n=188 in a study by Druss et al., older population (>65), or historic when interventions were not as readily available as in later years [20].

There are several potential causes to the observed increased long-term and 30-day mortality rates in schizophrenia patients with ACS. An important cause could be due to the lack of adjustment in different criteria (age, sex, smoking, previous PCI, other comorbidities, and timing of revascularization) between patients with and without schizophrenia. As summarized in Table 2, a recent study in 2020 by Attar R et al. described their findings on 1008 schizophrenic patients who experienced MI during 2018-2020. A remarkable expansion in mortality rate was noted in those patients when adjusted for age and sex only (HR 2.99, 95% CI 2.72-3.29). However, mortality-adjusted HR was reduced (aHR=2.38; 95% CI 1.84-3.09) when further factors were adjusted (smoking, comorbidities, previous PCI and coronary angiography, discharge medication, and treatment with coronary angiography and PCI) [16]. In support of this view, 1,145 schizophrenia patients with acute myocardial infarction (AMI) were studied by Hauck TS et al., who outlined a high mortality rate in those patients (HR 1.55; 95% CI, 1.37-1.77), which was reduced after adjustment for time-varying revascularization (aHR 1.38; 95% CI 1.20-1.58) [21]. Similar findings were seen in a study by Kugathasan P et al., which demonstrated a crude mortality HR of 18.61 (95%CI 16.31-21.24) in schizophrenia patients with ACS, which decreased to 15.47 (95% CI 12.62-18.96) after being adjusted for age, gender, calendar year, diabetes, hypertension, hyperlipidemia, and chronic obstructive pulmonary disease (COPD) [19]. To summarize, the overall trend shows us that there are confounding factors that increase the mortality of ACS in schizophrenic patients, such as smoking, BMI, demographic factors (income, urbanization), which may vary between studies. Another crucial factor is the overall trend in delay or not pursuing revascularization, which may vary by the health system.

Another possible cause of that increased observed trend is merging schizophrenia with other psychotic disorders and grouping patients into a broadly defined category of severe mental illness without being specific to only schizophrenia. This might participate in the described higher mortality rate in the cohort group when compared to the control group (patients with no psychotic disorders). Elevated one-year mortality after ACS was reported to be high in patients with psychotic disorders (schizophrenia, schizoaffective disorder, persistent delusional disorder, acute and transient psychotic disorders, and unspecified non-organic psychosis) by Chang W et al. (unadjusted OR 2.13; 95% CI 1.79-2.54). Even after being adjusted to age, sex, calendar-year period of index admission, and medical comorbidities, the mortality rate was persistently high (aOR 2.13; 95% CI 1.79-2.54; p< 0.001) [22]. Furthermore, differences in study designs and statistical methods involved might give a reason for the noted variability.

Not only were mortality rates higher, but also patients with schizophrenia tend to die at younger ages if they have ACS. A 15 to 25-year decrease in life expectancy in schizophrenic patients has been described in the literature [7-8]. This decrease can potentially be attributed to ACS complications. Westman J et al. looked specifically into this question and noted that death from ACS occurs at an earlier age in schizophrenia patients with a high early death (<60) in schizophrenia patients with ACS (MRR 5.39; 95% CI 4.84-6.00) [25]. This can possibly be attributed to patient factors and healthcare factors that will be discussed further in the next paragraphs. 

Morbidity of ACS in Patients With Schizophrenia

On their own, a diagnosis of either schizophrenia or ACS comes with a significant burden of morbidity that worsens when both conditions are combined. This combination is frequently observed with a higher risk of adverse outcomes, such as cardiovascular morbidities, stroke, and bleeding, noted in schizophrenia patients, as will be described below.

In our systematic review, we studied the main outcome of ACS in patients with schizophrenia. Major adverse cardiac events (MACE) are prominent outcomes. Two different studies by Attar R et al. and a third study by Protty MB et al. reported a notable rise in MACE rates in those patients when compared to psychiatric healthy patients with HR of (2.05; 95% CI 1.63-2.58), (1.62; 95% CI 1.45-1.81), and (1.20; 95% CI 1.09-1.31; p=0.05), respectively [16,23,26]. One important component of MACE post-ACS is stroke, which was reported to be higher in patients with schizophrenia. Specifically, stroke rates after ACS were studied by Attar R et al. and Protty MB. et al., revealing a statistically significant increase in the incidence of stroke in patients with schizophrenia versus those without, with HR (1.72; 95% CI 1.00-2.98; p<0.005), (1.51; 95% CI 1.15-1.99, p=0.0033) and (1.28; 95% CI 1.13-1.85; p<0.05), respectively [16,23,26].

Another serious complication of ACS after hospital discharge is bleeding, which is found to be higher in prevalence in patients with severe mental illness and more specifically in patients with a previous diagnosis of schizophrenia [26]. This could be attributed to the use of some medications. Antipsychotics (typical and atypical) through an unknown mechanism have been correlated to a higher risk of intracranial hemorrhage particularly in senior patients [32-33]. Moreover, antidepressants such as selective serotonin reuptake inhibitors (SSRI) have also been reported to have a side effect of more bleeding [34]. Two statistically significant studies reported contrasting results regarding bleeding after ACS [24,26]. A study was done by Protty MB et al in the United Kingdom showed an elevated bleeding rate (HR 1.35; 95% CI 1.15-1.58), whilst a reduced bleeding rate (OR 0.70, 95% CI 0.65-0.75) was noted in a national analysis of all hospitalization with AMI in the United States by Mohamed MO et al. Dissimilarity in healthcare system between the two countries (the United States and the United Kingdom) may contribute to that observed difference in the results.

Reassuringly, there were no differences reported in the reinfarction rates after ACS in patients with versus without schizophrenia as demonstrated by Attar R et al. (HR 0.88; 95% CI 0.73-1.07) and Protty MB et al. (HR 1.02; 95% CI 0.89-1.17) [23,26].

Possible Reasons to the Increased Mortality and Morbidity in Patients With Schizophrenia Post-ACS

Patient factors: The prevalence of cardiovascular disease and its risk factors are reported to be higher in patients with schizophrenia compared to the general population. For instance, the prevalence of diabetes mellitus has been observed to be at least two times as high as in the general population [10]. Also, the prevalence of metabolic syndrome in schizophrenia patients is up to four-fold the prevalence in the general population [10]. This may be mechanistically explained by pharmacological influence inferred by the use of medications such as antidepressants and antipsychotics, which have been linked to an increased risk of metabolic syndrome. These drugs interfere with glucose metabolism and thus weight control via interaction with target 5-hydroxytryptamine neuroreceptors. Thus, place patients on those medications are at an increased risk of ACS and its complication including all-cause bleeding, death, and stroke [35-41]. Obesity, anemia, COPD, peripheral arterial disease (PAD), and cardiomyopathy were reported to be more prevalent among patients with schizophrenia when compared to the general population [23].

**Figure 2:**
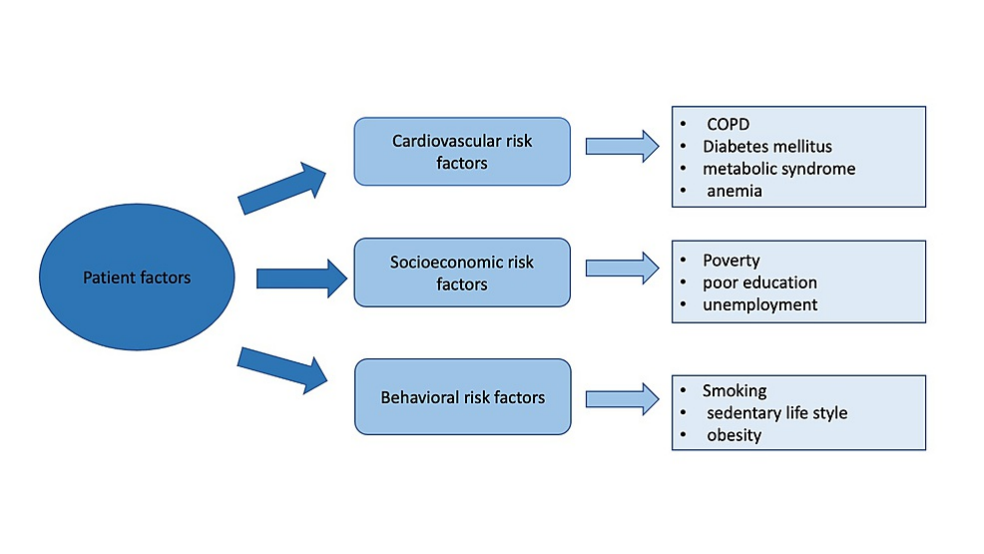
ACS risk factors in patients with schizophrenia ACS: acute coronary syndrome

Socioeconomic factors, such as poverty and lower educational attainment, are more prevalent in patients with schizophrenia compared to those without [17]. When these factors are combined with behavioral risk factors seen in patients with schizophrenia, such as substance abuse, tobacco smoking, sedentary lifestyle, and obesity, they may culminate in increased susceptibility to developing cardiac risk factors (like diabetes and metabolic syndrome) and not pursuing positive health choices [8,10]. Figure 2 highlights those factors, which may all contribute to the poor outcomes experienced by patients suffering from schizophrenia.

Healthcare factors: It is possible that patients suffering from schizophrenia receive suboptimal care at multiple levels in the healthcare system, which may lead to the persistently higher-than-expected mortality from ACS when compared to the general population [23,26]. Starting with primary prophylactic management, cardiovascular risk factors, documentation, and management are reported to be suboptimal in patients with schizophrenia [17]. Moreover, rates of revascularization post-ACS in patients with schizophrenia are almost half as much as those seen in the general population [17]. In addition, during the post-myocardial infarction (MI) care, there are further dissimilarities in providing care, with lower rates of cardiology specialist visits in the 30-days post-ACS in patients with schizophrenia when compared to those without (12.2% vs 20.1%, respectively) [30]. Other secondary prevention measures were also lower in schizophrenia patients, including the prescription of statins, beta-blockers, and anti-platelets [16].

**Figure 3:**
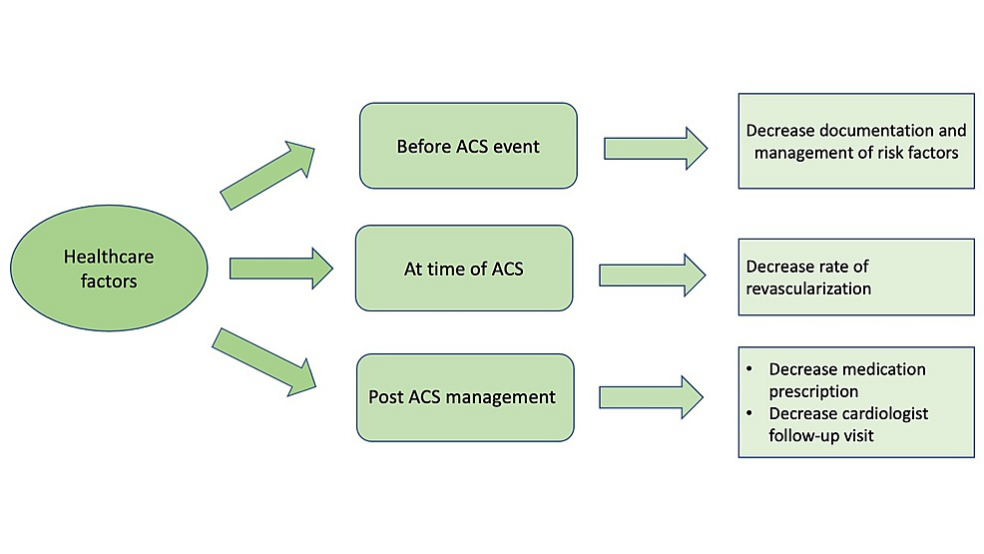
Health care factors that influence prognosis on schizophrenic patients with ACS diagnosis ACS: acute coronary syndrome

This suboptimal healthcare may be due to subconscious bias and stereotyping by health care providers when taking care of patients with severe mental illnesses overall and with schizophrenia specifically by deprioritizing those patients during ACS events [16]. There seems to be a preset perception that this population is poorly compliant, difficult to obtain medical or surgical treatment consents, and overall challenging to treat [27]. All of these perceptions may discourage healthcare staff from providing proper high-level care. These biases should be recognized and effectively managed when dealing with this vulnerable population with more attention given to ensuring proper follow-up arrangements. New guidelines should be placed to assist providers in the management of this cohort in the urgent setting of unstable medical and psychological issues. Figure 3 focuses its attention on the healthcare factors, which may lead to poor outcomes in patients with schizophrenia who suffer from ACS.

Limitations

All the included studies were retrospective studies, which may be limited by missing data, poor coding, or poor follow-up of participants. For this reason, a prospective study is needed to investigate mortality and morbidity following ACS in schizophrenia patients and to see how patient education about risk factors and lifestyle changes can affect those outcomes. Another limitation to this review is the exclusion of studies in languages other than English, which might have created an unintentional bias in our systematic review.

## Conclusions

In this systematic review, we explored the impact of schizophrenia on mortality and morbidity outcomes following an initial event of ACS. Death rates following ACS were reported to be higher in patients who were previously diagnosed with schizophrenia when compared to mentally healthy patients. Furthermore, schizophrenia patients suffering from ACS were reported to be at a greater risk of major complications after hospital discharge, including MACE, bleeding, and stroke compared to the general population. The causes for these findings are likely multifactorial, relating to patient factors (cardiovascular risk, behavioral, and socioeconomic factors), as well as healthcare factors (possible treatment bias, prejudice, and suboptimal care), which may contribute to the higher burden of mortality and morbidity in patients with schizophrenia. Patient education about risk factors, likely complications, and behavior modification may be employed alongside improving physician awareness of treatment inequality, management of mental health comorbidities, and encouraging social group and family support for schizophrenia patients. The effectiveness of any such measures needs to be assessed and addressed by future studies on this patient group.
